# Effect of Printing Parameters on the Thermal and Mechanical Properties of 3D-Printed PLA and PETG, Using Fused Deposition Modeling

**DOI:** 10.3390/polym13111758

**Published:** 2021-05-27

**Authors:** Ming-Hsien Hsueh, Chao-Jung Lai, Shi-Hao Wang, Yu-Shan Zeng, Chia-Hsin Hsieh, Chieh-Yu Pan, Wen-Chen Huang

**Affiliations:** 1 Department of Industrial Engineering and Management, National Kaohsiung University of Science and Technology, Kaohsiung 807618, Taiwan; shwang@nkust.edu.tw (S.-H.W.); tzen010@gmail.com (Y.-S.Z.); charlie820906@gmail.com (C.-H.H.); 2 Department of Fashion Design and Management, Tainan University of Technology, Tainan 71002, Taiwan; 3 Department and Graduate Institute of Aquaculture, National Kaohsiung University of Science and Technology, Kaohsiung 811213, Taiwan; 4 Department of Information Management, National Kaohsiung University of Science and Technology, Kaohsiung 824005, Taiwan

**Keywords:** 3D printing, FDM, PLA, PETG, mechanical properties, thermal deformation, sustainability

## Abstract

Fused Deposition Modeling (FDM) can be used to manufacture any complex geometry and internal structures, and it has been widely applied in many industries, such as the biomedical, manufacturing, aerospace, automobile, industrial, and building industries. The purpose of this research is to characterize the polylactic acid (PLA) and polyethylene terephthalate glycol (PETG) materials of FDM under four loading conditions (tension, compression, bending, and thermal deformation), in order to obtain data regarding different printing temperatures and speeds. The results indicated that PLA and PETG materials exhibit an obvious tensile and compression asymmetry. It was observed that the mechanical properties (tension, compression, and bending) of PLA and PETG are increased at higher printing temperatures, and that the effect of speed on PLA and PETG shows different results. In addition, the mechanical properties of PLA are greater than those of PETG, but the thermal deformation is the opposite. The above results will be a great help for researchers who are working with polymers and FDM technology to achieve sustainability.

## 1. Introduction

Due to global competition, the mass customization of products, and the long molding cycle of traditional manufacturing methods, the manufacturing industry is under more pressure to seek the advantages of new processes that can cope with small batches and rapid manufacturing. In recent years, 3D printing technology in the medical, manufacturing, and engineering fields has developed rapidly [1,2]. This technology is also known as Additive Manufacturing (AM) technology, which is based on incremental layer-by-layer manufacturing [3]. It is not only directly fabricated from 3D digital models but it also does not require any fixtures or specific tools. It can manufacture complex structures and print multi-materials quickly, compared to any other method. AM technology can be divided into three basic groups, namely those that are solid-based, powder-based, and liquid-based. The three most commonly used techniques are Fused Deposition Modeling (FDM) [4,5], Selective Laser Sintering (SLS) [6,7], and stereolithography (SLA) [8,9], where FDM is solid-based, SLS is powder-based, and SLA is liquid-based.

FDM is the first choice of AM technology for polymer and composite materials, due to its flexibility, higher printing speed, low cost, high strength and toughness, non-toxicity, and the diversity of materials, in comparison with other AM technologies. A spool of thermoplastic material, in the form of filaments, is most commonly used in FDM technology to produce the 3D parts. Firstly, the thermoplastic materials (e.g., ABS, PLA, PC, PS, Nylon, and PET) are melted by a heated liquefier to extrude the filaments through a nozzle. The thermoplastic filaments are in a semi-solid state during the extrusion. After this, the nozzle can move in an X–Y direction to deposit semi-solid filaments onto the plane, and then it cools, solidifies, and integrates with the surrounding filaments. After a layer is deposited, the build platform is moved downward in a Z-direction, and another layer is deposited onto the previous layer. Therefore, FDM can manufacture any complex geometrical and internal structures and has become one of the most popular AM technologies. Nowadays, the technology of FDM is widely applied in many industries, such as the biomedical, manufacturing, aerospace, automobile, industrial, and building industries [10,11,12,13]. However, FDM is deposited layer by layer, so the adhesion between the layers is weak, and its mechanical properties are poor, in comparison to injection molding. In addition, it is a complex process and many process parameters need to be adjusted, which will influence the quality and properties of the parts. Therefore, the quality and properties of the parts can be improved by setting a reasonable range of process parameters.

In order to avoid the loss of printing materials and time, as noted by other researchers, this study discusses the effect of printing parameters on the thermal and mechanical behavior of PLA and PETG, and printing parameters include the temperature and the speed of printing. The recommended printing temperature for PLA ranges from 180 °C to 210 °C, while the recommended printing temperature for PETG ranges from 215 °C to 235 °C.

## 2. Literature Review

Over the past several years, researchers have explored the influence of the process parameters of printing on the key metrics of FDM technology to improve the quality and the properties of the parts, in order to guarantee a reliable structural performance and to decrease the building cycle.

Jiang et al. [14] explored the tensile strength and the fiber distribution of PLA, ABS, PETG, and Amphora by adding corban fiber, with the raster angle of the printing being 0°, 45°, ±45°, and 90°, respectively. The results showed that PETG had the highest tensile strength after adding corban fiber. In particular, when the raster angle was set at 0°, the tensile strength and the Young’s modulus increased to 48.2% and 313.2%, respectively. Mansour et al. [15] explored the compressive strength, nano-indentation, and modal parameters of PETG by adding a 20% mixture of carbon fiber, and the results showed that the specimen’s compressive strain decreased by 66% after adding a mixture of carbon fiber, while the modal and hardness increased to 30% and 27%, respectively, and the damping and loss factors also decreased from 17.3% and 15.3% to 13.8% and 12.39%, respectively. Santana et al. [16] explored the differences among the mechanical properties of PLA and PETG, based on FDM and injection molding technology, and the results showed that PLA has higher stiffness and tension than PETG; however, PETG has better thermal degradation resistance and thermal stability than PLA, and, after the FDM process, the tensile strength of PLA and PETG increases by 24% and 18%, respectively. Because the homoscedasticity of PETG is lower than that of PLA, PETG should have better printing stability. Rajpurohit et al. [17] explored the effect of the angle and width of the grid and layer height on PLA, and the results showed that, when the angle of the grid is 0°, it has the highest tensile strength, and that the tensile strength can be improved by enhancing the width of the grid and reducing the layer height. Valerga et al. [18] analyzed the effects of the printing temperature, humidity, and the color of the wire on the mechanical properties, based on PLA, and the results showed that a higher printing temperature will enhance the deviation of the product size and reduce the tensile strength, while the deviation will increase substantially when the printing temperature is over 200 °C. As far as the mechanical properties are concerned, the printing temperature should be set at 220 °C. Vinyas et al. [19] studied composites of PLA, such as PLA +30% nylon glass fibers, PLA +10% carbon fibers, and a PLA + PET-G polymer blend, after analyzing the tensile strength and heat-resistance, and the results showed that PLA + 10% carbon fibers had the highest tensile strength. Meanwhile, PLA +30% glass fibers could provide excellent thermal stability and can be used where primary importance is given to thermal stability over the mechanical strength. Singh et al. [20] divided ABS, PLA, and High-Impact Polystyrene (HIPS) into three slices and changed the order of printing to produce test specimens. They analyzed the tensile strength by arranging the different materials, and the results showed that the tensile strength of the specimens increased to 10.78 Mpa by printing in three layers, with the first layer in ABS, the second layer in PLA, and the last layer in HIPS. Yao [21] explored the effects of different layer thicknesses on the UTS of PLA, including 0.1, 0.2, and 0.3 mm, with a raster angle of 0°, 15°, 30°, 45°, 60°, 75°, and 90°, respectively. The results showed that the raster angle affected the UTS significantly, and the discrepancy between the specimens of 0.1 mm–0° and 0.1 mm–90° reached 52.29%. The present paper shows the effect of printing parameters on the mechanical properties of the materials. The researchers used different methods, including adjusting the parameters, adding other fibers, changing the process, and so on, to improve the efficiency, productivity, and mechanical characteristics of the printing objects. Barrios et al. [22] explored the parameters needed to ensure that a PETG wire had the best Ca value, based on FDM, including the layer height, nozzle temperature, process speed, the acceleration of the process speed, and the flow of material. After analyzing them using the Taguchi method and Analysis of Variance (ANOVA) methods, the results showed that the acceleration of the process speed and the flow of the material had the strongest influence on the quality parameters of PETG. Guessasma et al. [23] explored the effect on the tensile strength of PETG by setting the nozzle temperature at 210 °C~250 °C, and the results showed that the PETG wire must be printed at a temperature of over 230 °C; otherwise, the material cannot be pasted onto the platform. When the nozzle temperature is set at 250 °C, the porosity has a maximum value of 2% and the average roughness is approximately 100 μm. In addition, the FDM process will reduce the tensile strength and stiffness of PETG by over 40%, and the elongation will also decrease drastically. The present paper on PETG shows that it has better heat resistance than PLA, and, therefore, many producers are expected to use PETG in the future, in order to compensate for the disadvantages of PLA. Many researchers have explored a large amount of data on PLA, based on FDM, and if the parameters of PLA and PETG could be cross-referenced, the printing parameters of PETG could be evaluated by the data history of PLA in the future. Bakradze et al. [24] present a heuristic procedure for determining the key processing parameters (PPs) of PA and ABS; the results showed that it is possible to reduce the optimization time down to several hours, as well as to reduce the amount of consumed feedstock material. Tensile tests revealed a strong effect of the amorphous and semi-crystalline nature of the polymer on the results of optimization.

## 3. Materials and Methods

### 3.1. 3D Printing Machine and Printing Materials

The 3D printer used in this study is the X1E produced by INFINITY 3DP Co., Ltd. (Kaohsiung, Taiwan). It uses a 1/32 micro-stepping motor, so that the slide rail is more stable when moving. This study mainly compared the material properties of PLA and PETG. Therefore, the size of the selected nozzle was 0.4 mm to reduce the complexity of the study, and the main structure and the support structure were printed by the same nozzle. The printing temperature of the nozzle can reach up to 300 °C. The temperature of the platform can reach up to 110 °C. The specifications of the 3D printer are shown in Table 1. The materials that were tested were polylactic acid (PLA) and polyethylene terephthalate glycol (PETG) from the Min-Yau Information Co., Ltd. (New Taipei, Taiwan), which are both strongly thermoplastic. Before printing, the PLA and PETG materials were placed in a humidity control box to keep them dry. The specifications of the printing materials are shown in Table 2.

### 3.2. Printing Procedure and Parameters

**Figure 1:**
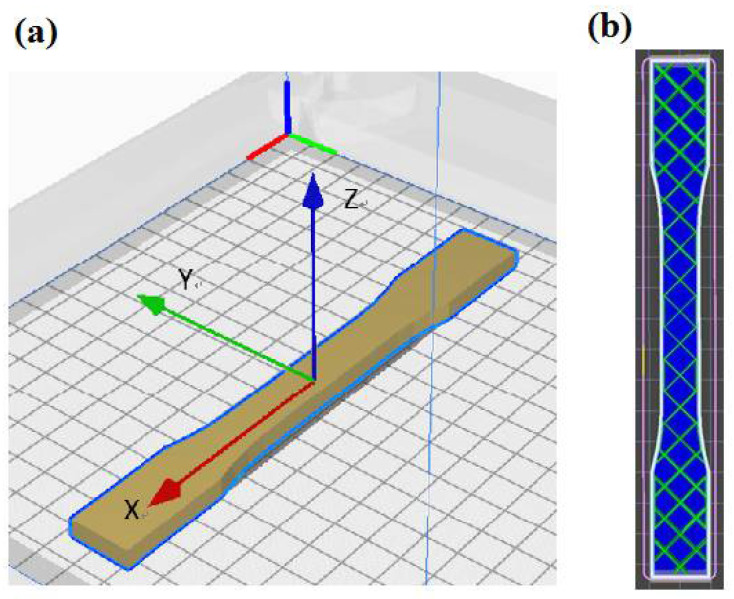
(**a**) Definition of the specimen orientations; (**b**) dimensions and trajectories used for monolayer samples.

Firstly, SolidWorks software was used to create a 3D virtual geometry of the test specimens. Since most slicing software accepts image files in .obj and .stl format, the SolidWorks file was converted to .stl format and was exported into the slicing software after creating the virtual test specimens. The slicing software uses KISSlicer software because the system of this software has more parameters that can be adjusted, in order to print more detailed test specimens. Since the melting and freezing points of the two printing materials in this experiment were different, if PETG was printed at the same printing speed and temperature as PLA, it would increase the probability of the object warping and threads pulling or peeling off the printing platform. Therefore, the selected printing speed and temperature range were different, as shown in Table 3. The fixed parameter conditions included an infill density of 20%, a raster angle of 45°, a layer thickness of 0.2 mm, and a platform temperature of 25 °C. The X, Y, Z labels used for the specimen orientation are shown in Figure 1. The quality of a 3D-printed object depends on the quality of the initial 3D model of the object; therefore, the image acquisition step is essential to the quality of the 3D-printed object [24,25,26]. The three different requirements of printing quality are: (i) low surface roughness, (ii) good mechanical properties, and (iii) short printing time. This study discusses the mechanical properties of printing quality.

### 3.3. The Tensile, Compression, and Bending Test

To evaluate the tensile, compression, and bending properties of the specimens, the test was performed using the QC-H51A2 universal testing machine, the specifications of which are shown in Table 4. The specimens were tested at a rate of 5 mm/min and at room temperature, kept at 23 °C. The stress and strain data of the test, with a force of 100 kN, were recorded by the built-in program. The data obtained from the tests were analyzed by using Excel software to determine the values of Young’s modulus and the ultimate strength of the specimens. The tensile specimens were prepared according to ASTM D638 standard testing, the compression specimens were prepared according to ASTM D3410 standard testing, and the bending specimens were prepared according to ASTM D790 standard testing.

### 3.4. The Thermal Deformation Test

The thermal deformation test was performed to evaluate the thermal deformation properties of the specimens, using the QC-654 heat deflection tester. The specimen was placed on the Charpy of the structure, and the main function was a test of the center position of 0.445 MPa or a bending stress of 1.82 MPa. The test environment temperature was increased by 2 °C/min, in order to measure the specimen’s center of deformation, with a 0.25 mm temperature value as the deformation temperature. This test is suitable for material at 27 °C, which is still a hard material. The thermal deformation specimens were prepared according to ASTM D648 standard testing, while the specifications of the QC-654 heat deflection tester are shown in Table 5.

## 4. Results and Discussion

**Figure 2:**
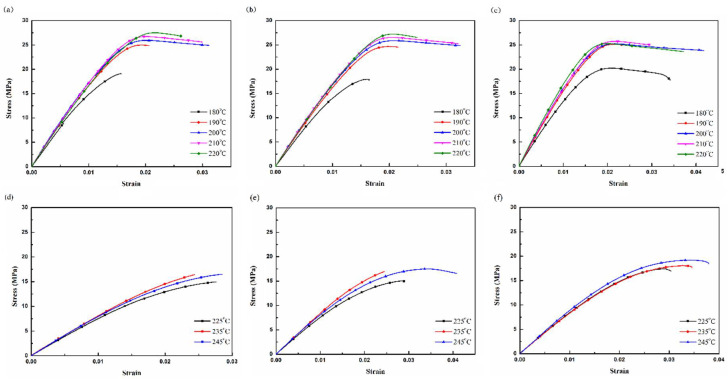
Stress-strain curves of tensile tests (**a**–**c**) 45 to 35 mm/s of printing speeds for PLA specimens, and (**d**–**f**) 35 to 25 mm/s of printing speeds for PETG specimens.

Each graph of mechanical and thermal properties was obtained by the average value of four replicates over five experiments. Figure 2 presents the stress–strain curves of the tensile tests. It can be seen that the material properties of PLA are similar to an elastic–plastic response, the material properties of PETG are similar to a brittle response, and the break strain increases with the decreasing printing speed [27]. The stress–strain curves of PLA are similar for printing temperatures up to a strain value of around 0.01, except for a printing temperature of 180 °C (Figure 2a–c), at which the PLA material has not completely reached melting point. The stress–strain curves of PETG are similar for all printing temperatures (Figure 2d–f), because PETG melts completely at a printing temperature of over 225 °C. It can be seen that the tensile strength is equal to the break stress and seems to be independent of the printing temperature.

**Figure 3:**
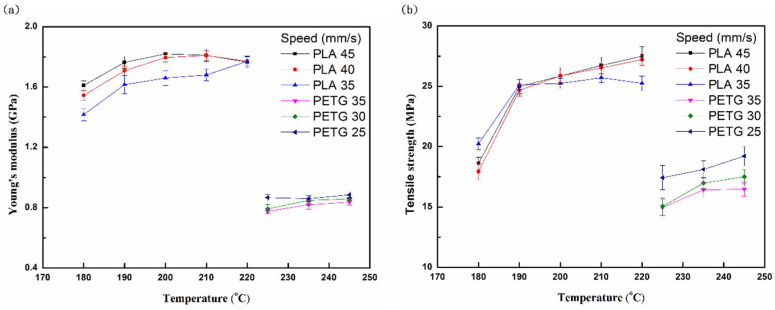
(**a**) Young’s modulus and (**b**) tensile strength of PLA and PETG specimens after tensile tests at different printing temperatures and printing speeds.

Figure 3 shows the tensile properties (Young’s modulus and strength) of PLA and PETG specimens at different printing temperatures and speeds. It can be seen that the low fluidity and high viscosity of the polymer melt at a low temperature can result in poor bonding and high porosity between the lines and layers of the molten polymer [18,28]. Therefore, the tensile properties are poor at low printing temperatures. As the printing temperature increases, the viscosity of the PLA and PETG melts decreases, which results in increased fusion between the polymer fuse and the layers, and the porosity decreases. Therefore, the tensile properties are increased accordingly and those for PLA are higher than those for PETG. The scanning electron microscope (SEM) micrographs of the references [29] taken from fracture planes support the above reasoning. The tensile properties of PLA increase when the printing speed increases, because the heat dissipates rapidly and it has a low filling rate, as the fusion between the layers improves. The tensile properties of PETG increase when the printing speed decreases, and because its heat dissipation is slow, it has low porosity.

**Figure 4:**
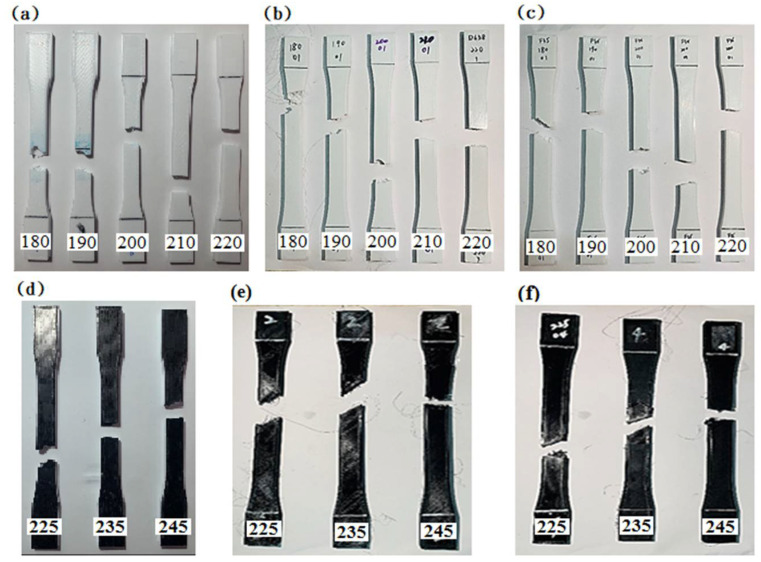
Images of (**a**–**c**) PLA specimens and (**d**–**f**) PETG specimens after tensile tests. The printing speed decreases from (**a**) to (**f**): 45, 40, 35, 35, 30, and 25 mm/s.

Figure 4 shows images of the PLA and PETG specimens at different printing temperatures after the tensile tests. The printing temperatures increase from left to right. Two failure modes are defined, namely an interlayer failure mode and an in-layer failure mode [30,31]. It can be observed that there are different stress distributions at each printing temperature and that the failures follow the weaker areas of the pattern. The layer bonds increase with the increasing printing temperature. An interlayer failure mode occurred when the layer bonds of the specimens were insufficient, and it failed along the 45 line of the upper layer. In addition, most of the failure planes of the in-layer failure mode were approximately perpendicular to the material layer. These results agree with those obtained in previous studies.

**Figure 5:**
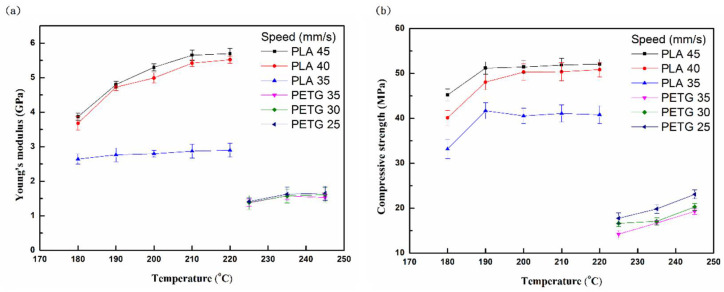
(**a**) Young’s modulus and (**b**) compressive strength of PLA and PETG specimens after compression tests at different temperatures and printing speeds.

Figure 5 shows the compression properties (Young’s modulus and strength) of the PLA and PETG specimens at different printing temperatures and speeds. From all the results, it was found that the values of the compression properties increase with the increasing printing temperature. This is due to the decrease in the contact stress at the line and line contacts. It was observed that there was a minimal effect on the compression properties of PETG with respect to the printing speed. The stress in compression largely exceeded that measured in the tension, for any given strain. A strong tension and compression asymmetry was observed [32,33]. This was caused by the residual stress, which was due to the cooling down of the specimen to room temperature. In addition, the compression properties of PLA were greater than those of PETG, similar to their tensile properties.

**Figure 6:**
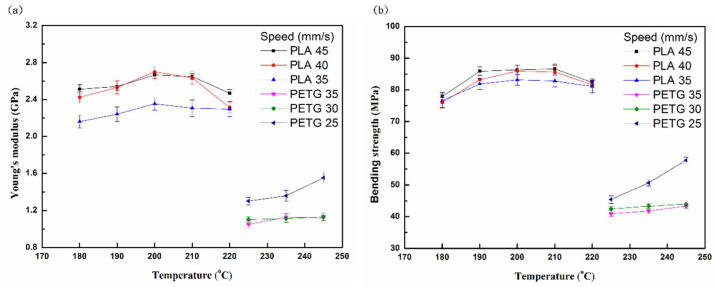
(**a**) Young’s modulus and (**b**) bending strength of PLA and PETG specimens after bending tests at different temperatures and printing speeds.

Figure 6 shows the bending properties (Young’s modulus and strength) of PLA and PETG specimens at different printing temperatures. The most remarkable finding regarding the graphics related to the strength is that the bending value is considerably higher than in the compressive and tensile strengths because of the residual stress [34]. The graphics show that the compressive Young’s modulus is higher than that of the bending Young’s modulus and the tensile Young’s modulus. The tensile properties are the lowest, regardless of Young’s modulus and strength. In addition, the bending properties of PLA are greater than those of PETG, similar to their tensile properties and compression properties [35].

**Figure 7:**
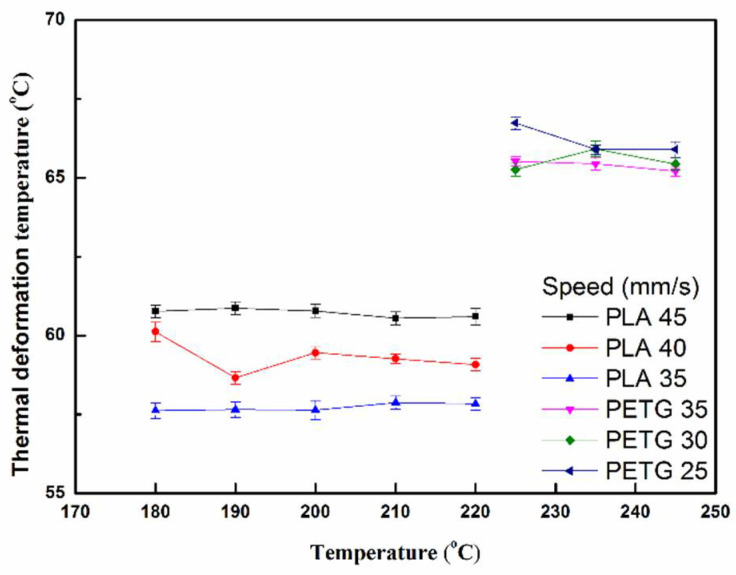
Thermal deformation temperature of PLA and PETG specimens at different printing temperatures and printing speeds.

Figure 7 presents the thermal deformation of the PLA and PETG specimens at different printing temperatures. It can be seen that the PLA specimens (45 mm/s) have a higher thermal deformation temperature than the PETG specimens (40 and 35 mm/s). The PETG specimens (25 mm/s) have a higher thermal deformation temperature than the PLA specimens (30 and 35 mm/s). The result shows that PETG performs better than PLA in terms of heat resistance, which may be because the glass transition temperature of PETG is higher than that of PLA [16].

## 5. Conclusions

The effect of the printing temperature and printing speed on the PLA and PETG constructed by FDM was investigated. The mechanical properties (tensile, compression, and bending) and thermal properties of the specimens were tested. The main conclusions are as follows:

1. PLA and PETG materials exhibit obvious tensile and compression asymmetry, and the compressive stress exceeds the tensile stress.
2. As the printing temperature increases, the mechanical properties of the PLA and PETG materials increase.
3. As the printing speed increases, the mechanical properties of the PLA material increase, but the mechanical properties of the PETG material decrease.
4. The mechanical properties of PLA are greater than those of PETG, regardless of the Young’s modulus and strength, but the opposite is the case for the thermal deformation.
5. In this article, despite the PLA and PETG being below the 20% infill density conditions, the compressive strength is higher than the tensile strength, but lower than the bending strength, with a bending Young’s modulus that is higher than the tensile Young’s modulus but lower than the compressive Young’s modulus.

## Figures and Tables

**Table 1:** The specifications of the 3D printer.

Model	X1E
Physical dimensions	(w) 40 cm × (d) 22 cm × (h) 46 cm
Maximum printing area	(w) 21 cm × (d) 21 cm × (h) 24 cm
Print layer height	0.04~0.32 mm
Wire diameter	Φ1.75 mm
Nozzle diameter	0.2, 0.4, 0.6 mm
Platform temperature	~110 °C
Nozzle printing temperature	~300 °C
Cooling method	4.5 cm turbo fan
Motor drive	1/32 micro-stepping motor (8825 driver chip)

**Table 2:** The specifications of the printing materials.

Name	Specification	
Material	PLA	PETG
Color	Snow white	Matt black
Wire diameter	1.75 ± 0.05 mm	1.75 ± 0.05 mm
Weight	800 g	800 g
Recommended printing temp	180~210 °C	215~235 °C
Recommended printing speed	30~50 mm/s	30~50 mm/s

**Table 3:** Process parameter conditions of the printing material.

	Content	Name	Range	
Project				
Controlling factor		Material	PLA	PETG
		Printing speed	35~45 mm/s	25~35 mm/s
		Printing temperature	180~220 °C	225~245 °C
Fixed factor		Infill density	20%	
		Raster angle	45°/−45°	
		Printing pattern	rectilinear	
		Layer thickness	0.2 mm	
		Nozzle diameter	0.4 mm	
		Platform temperature	25 °C	

**Table 4:** The specifications of the QC-H51A2 universal testing machine.

Model	H51A2
Capacity	100 kN
Stroke	1100 mm (without fixture)
Space	Ø550 mm
Load resolution	1/10,000 (maximize 1/200,000)
Displacement resolution	0.001 mm
Motor	Servo motor
Speed	0.003~375 mm/min
Height	2200 mm
Weight	800 kgf
Sampling rate	500 Hz (Max.)
Current	15 A

**Table 5:** The specifications of the QC-654 heat deflection tester.

Model	QC-654
Temperature	Room temperature ~300 °C
Temperature control	Temperature rise speed 2 °C/min
Weight sets	Basic weight support load is 3 N. Each test fixture will enclose the below quantities: 0.1 N × 1, 0.2 N × 2, 0.5 N × 1, 1 N × 1, 2 N × 2, 5 N × 1, 10 N × 1
Deformation	0.01~10 mm
Span	100 mm (max.) adjustable 50 mm
Radius of support	Camber R angle 3.0 mm
Test quantity	Mechanical gauge 0.01~10 mm Electronic gauge 0.001~10 mm PLC control 0.001~10 mm Set: 1, 2, 3, 6 set
